# Health care system and patient costs associated with receipt of minimally adequate treatment for depression and anxiety disorders in older adults

**DOI:** 10.1186/s12888-022-03759-9

**Published:** 2022-03-10

**Authors:** Catherine Lamoureux-Lamarche, Djamal Berbiche, Helen-Maria Vasiliadis

**Affiliations:** Faculty of Medicine and Health Sciences, Campus de Longueuil – Université de Sherbrooke; Centre de recherche Charles-Le Moyne, 150 Place Charles-Le Moyne, Quebec J4K 0A8 Longueuil, Canada

**Keywords:** Minimally adequate treatment, Depression, Anxiety disorders, Health care costs, Patient costs, Older adults, Canada

## Abstract

**Background:**

Depression and anxiety disorders in older adults are associated with a great burden. Research has shown that less than 50% of adults receive adequate treatment in primary care settings for these disorders. Rare are the studies however assessing adequate treatment in older adults and associated costs from the societal perspective. Given the episodic nature of common mental disorders, this study aims to assess the three-year costs from a restricted societal perspective (including health system and patient perspectives) associated with receipt of minimally adequate treatment for depression and anxiety disorders in older adults consulting in primary care.

**Methods:**

This primary care cohort study included 358 older adults aged 65 years and older with either a self-reported or physician diagnosis of depression or an anxiety disorder covered under Quebec’s public drug plan. Receipt of minimally adequate treatment was assessed according to Canadian guidelines and relevant reports. Outpatient and inpatient service use, medication costs and physician billing fees were obtained from provincial administrative databases. Unit costs were calculated using provincial financial and activity reports and relevant literature. A propensity score was created to estimate the probability of receiving minimally adequate treatment and the inverse probability was used as a weight in analyses. Generalized linear models, with gamma distribution and log link, were conducted to assess the association between receipt of minimally adequate treatment and costs.

**Results:**

Overall, receipt of minimally adequate treatment was associated with increased three-year costs averaging $5752, $536, $6266 for the health system, patient and societal perspectives, respectively, compared to those not receiving minimally adequate treatment. From the health system perspective, participants receiving minimally adequate treatment had higher costs related to emergency department (ED) (difference: $457, *p* = 0.001) and outpatient visits (difference: $620, *p* < 0.001), inpatient stays (difference: $2564, *p* = 0.025), drug prescriptions (difference: $1243, *p* = 0.002) and physician fees (difference: $1224, *p* < 0.001). From the patient perspective, receipt of minimally adequate treatment was associated with higher costs related to loss of productivity related to ED (difference: $213, *p* < 0.001) and outpatient visits (difference: $89, *p* < 0.001).

**Conclusions:**

Older adults receiving minimally adequate treatment for depression and anxiety disorders incurred higher societal costs reaching $2089 annually compared to older adults not receiving minimally adequate treatment. The main cost drivers were attributable to hospitalizations and prescription drug costs.

## Introduction

With the aging of the Canadian population, the number of individuals with mood and anxiety disorders will increase by 23% in the next 20 years reaching 4.9 million Canadians [1]. Recent data from the Global Burden of Disease Study showed that 7.5 and 3.4% of years lived with disability (YLD) were attributed to depressive and anxiety disorders [2]. Annual excess health care costs (i.e. ambulatory visits, hospitalizations, fees paid to physicians and outpatient medications covered under the public drug plan) associated with depression and anxiety disorders in older adults in Canada were estimated at $1.5 Billion [3, 4]. A population-based study also showed increased costs associated with the presence of mental disorders in older adults with chronic conditions reaching $600 per person [5]. Despite the important burden associated with these common mental disorders (CMD), population studies show that less than 50% of patients receive adequate treatment [6–8]. In one of the rare studies on older adults, only 57 and 38% received minimally adequate treatment for depression and anxiety [9]. Further, individuals receiving minimally adequate treatment for CMD were on the one hand more likely to have a persistent disorder and on the other, to report improvement in satisfaction with life over a three-year period [9]. These estimates highlight the important societal burden associated with common mental disorders in older adults and the need for improving access to mental health care.

The association between receipt of adequate treatment for depression and anxiety disorders and health care costs for outpatient visits, hospitalizations and outpatient medications in adult populations have been inconsistent [10–13]. In the Netherlands, receipt of guideline-concordant care for depression and anxiety disorders was associated with higher costs reaching 1035 EUR annually [12]. In Canada, a health administrative registry study showed that adults with depression receiving the lowest and highest levels of guideline-concordant care, based on antidepressant type, dose and duration, incurred the highest costs suggesting a V-shape relationship [10]. In a representative sample of general population older adults, the use of antidepressants was associated with increased health care and patient costs averaging $1980 CAD [14].

To our knowledge, no study to date has assessed the costs associated with receipt of minimally adequate treatment for depression and anxiety disorders in older adults with either a self-reported or a physician diagnosis which overcomes the problem that less than one in two older adults consult for their mental health problems [15, 16]. Difficulties in measuring and assessing guideline-concordant treatment for depression using administrative data have also been underlined [17]. Rare are the studies that have measured receipt of minimally adequate treatment for depression and anxiety disorders from a broader definition that includes services such as psychotherapy using self-reported and administrative data. Most studies did not consider costs from the societal perspective, which also includes the health care system and patient perspectives. Further, costs associated with adequacy of treatment have been studied on shorter periods between 6 and 12 months [10–12]. Given that the length of an episode of depression and anxiety average 23 months in older adults [18], it is important to assess the long-term costs associated with an episode. Given the gaps in the literature, this study aimed to assess the three-year costs from the societal perspective, including health care and patient costs, associated with receipt of minimally adequate treatment for depression and anxiety by linking health administrative to self-reported health survey data in a patient population of older adults. The findings can inform decision makers for better allocation of human and financial resources to provide quality mental health care for older adults with depression and anxiety disorders followed in primary care.

## Methods

This primary care cohort study focused on 358 older adults with depression or an anxiety disorder covered by the provincial public drug plan and participating in the longitudinal *Étude sur la Santé des Aînés* (ESA)-Services study. French-speaking older adults aged ≥65 years (*n* = 1765) were recruited between 2011 and 2013 in primary care clinics in one of the largest health administrative regions in Quebec, Canada. At the time of the study, 1,469,000 inhabitants lived in this region, which represents almost 20% of the population of Quebec [19].

A detailed description of the longitudinal ESA-Services study has been published elsewhere [20]. Briefly, older adult patients in the waiting room of participating physicians received a pamphlet describing the study objectives. Interested participants filled out their contact information and were called by the study coordinator. Face-to-face interviews were conducted within the month by trained research assistants. Consent was obtained at the beginning of the interview. Participants with moderate to severe cognitive impairment, scoring lower than 22 on a French-Canadian translation of the Mini-Mental State Examination (MMSE) adapted for at home interviews [21, 22], were excluded from the analyses. Participants also gave consent for access to their health administrative data during the three years prior and following the interview. In Quebec, all citizens are covered under the provincial public health insurance plan (Régie de l’Assurance Maladie du Québec [RAMQ]) that provides outpatient and inpatient medical care throughout the province at no costs. Further, all citizens are required to be covered by an insurance for outpatient medications. Individuals not covered under a private insurance are automatically covered by the province’s public drug plan.

Participants with either a self-reported or physician diagnosis of depression or an anxiety disorder (CMD) were included in the analytic sample. Self-reported CMD in the last six months were aligned with DSM-5 criteria [23] with the exception of the criteria on functional impairment and included major depression, specific phobia, social phobia, panic disorder, agoraphobia and generalized anxiety disorder. Physician diagnoses of CMD in the six months preceding and following the interview were captured in the administrative databases using the following International Classification of Diseases (ICD) 9th and 10th revisions codes: 300.0, 300.2, 311.0; 311.9; 300.4; F32; F33; F34.1, F40, F41 [10, 24–27]. The research ethics committees of the CISSS Montérégie-Centre and the CIUSSS de l’Estrie-CHUS approved the longitudinal ESA-Services study.

### Measures

#### Minimal adequate mental health care from an epidemiological perspective

Adequacy of mental health care for CMD was measured at baseline using minimal quality indicators as it was not possible to account for clinical profiles, history of treatment and patient preferences [28]. The definition was based on Canadian guidelines and relevant publications [6, 29–37]. Receipt of minimally adequate treatment for depression was based on the presence of an antidepressant prescription and four follow-up visits with a general practitioner (GP) within three months following the diagnosis or the dispensing of the first antidepressant prescription or, eight individual or group psychotherapy sessions within a twelve-month period [30, 31, 33, 35, 37]. For anxiety disorders (with the exception of specific phobia), receipt of minimally adequate treatment was based on the dispensing of an antidepressant, anxiolytic or another recommended medication, and four follow-up visits with a GP within three months of the diagnosis or first dispensing of drug, or seven individual or group psychotherapy sessions within a twelve-month period [6, 29, 34]. Since pharmacological treatment is not recommended for specific phobia, minimally adequate treatment for this anxiety disorder was defined as the presence of five psychotherapy sessions within a twelve-month period [32]. Participants receiving minimally adequate treatment for depression or an anxiety disorder were categorized as receiving minimally adequate treatment.

#### Study variables

This study was based on Andersen’s conceptual framework on health care-seeking behaviors and related health outcomes [38], which considers individual and health system predisposing, enabling and need factors, measured at baseline. Individual predisposing factors included age, sex (male/female), education (0–7 years/≥8 years) and marital status (married/not married).

Individual enabling factors included area level material and social deprivation, social support, quality of life, patients’ perceptions regarding mental health treatment,  confidence in doctor to help with emotional problems and patient self-reported perceived adequacy of care. Area level material and social deprivation indexes were based on information published by the Quebec National Public Health Institute and categorized into quintiles (1 to 5, lowest to highest deprivation) [39]. These indexes are based on the following six indicators: (1) the proportion of individuals aged 15 years and older who do not have a secondary diploma or certificate, (2) who are employed, (3) who are not married or living with a partner and 4) who are living alone, 5) the average income for individuals aged 15 year and older, and (6) the proportion of single-parent families [39]. Social support was measured by the presence of three different types of support including emotional, instrumental and informational with scores ranging from 0 to 3. A higher score indicates higher social support [40]. Self-reported quality of life in older adults [41] included health-related quality of life (HRQOL), measured with the aid of a visual analog scale ranging from 0 to 100 (worst to best health state) and satisfaction with life assessed with an adapted French version of the Satisfaction with Life Scale (5–25) [42, 43]. Higher scores on these instruments indicate better HRQOL and satisfaction with life. Participants’ beliefs regarding mental health treatment, confidence in doctor to help with emotional problems and patient self-reported perceived adequacy of care were assessed with the following questions on a five-point rating scale: 1) In your opinion, do people who see a health professional to resolve their emotional or nervous problems are really helped? [not at all to absolutely]; 2) How would you say the doctor you met is the type of professional who can help you with an emotional issue? [not at all to absolutely] and 3) Would you say that the frequency of medical visits with your doctor to discuss your emotional symptoms is adequate (respond to your needs)? [not at all to absolutely]. The type of primary care practices participants were recruited in was considered as an enabling health system factor. Primary care practices included small private clinics with less than three physicians and large clinics (private clinics with at least three physicians, family medicine groups and local community service centers).

Individual need factors included cognitive functioning measured with a French version of the MMSE adapted for at home interviews (22–30) [21, 22], number of current chronic physical disorders (0–17), number of daily hassles (0–32) [44], self-reported psychological distress measured using the 10-item Kessler Psychological Distress Scale (K10) (10–50) [45] and past-month anxiety symptom severity based on a French translation of the 7-item Generalized Anxiety Disorder (GAD-7) questionnaire (0–21) [46]. Higher scores on these scales indicate more severe symptoms.

### Cost variables

The cost analysis was based on a restricted societal perspective, which included costs estimated from the patient and health system perspectives measured during the three-year period post interview, and this following economic evaluation guidelines [47–49] and published methods [3, 14, 50]. Costs measured from the health care system perspective included those for servi c es incurred during a hospital stay in general and psychiatric wards (per diem cost) and day surgery (cost per day surgery), an outpatient and emergency department (ED) visit (cost per visit), a mental health outpatient visit (annual cost p er patient in addition to cost per visit), medical billing fees paid to physicians and costs related to drugs dispensed in community settings (cost of prescription paid by the provincial drug plan). Medical ambulatory consultations were identified from the *Régie de l’Assurance Maladie du Québec* (RAMQ) database. Physician fees paid out for medical acts and consultations were captured in the RAMQ’s medical registry. The presence of a hospitalization and one-day surgery was assessed using the *Maintenance et exploitation des données pour l’étude de la clientèle hospitalière* (MED-ECHO) provincial database, which includes individual information on length of stay, admission and discharge diagnoses, medical consultations and acts received. Mental health outpatient visits and inpatient stays were determined by the presence of a mental health diagnosis using ICD-9 and ICD-10 codes [26, 27] in the RAMQ and MED-ECHO databases. The RAMQ pharmaceutical services registry includes information on prescriptions dispensed to all residents covered under the provincial drug plan, the cost of the drug, pharmacist dispensing fee and cost paid by beneficiaries.

Unit costs were calculated from information obtained in the financial and activity reports (AS-471) [51] of the Ministry of Health and Social Services using the direct allocation method [49]. These reports, which include information from the financial records of each health facility in Quebec, allow the calculation of the average cost by activity center [52]. Data from the literature were also used to estimate overhead costs such as those related to security and maintenance [3], opportunity costs for lands and facilities [53] and depreciation costs for buildings [54]. Briefly, 9.7% of total health care costs were added to unit costs for ED, inpatient and outpatient services to account for security and maintenance of equipment and buildings [3] and 10% for the depreciation of buildings [54]. To account for the opportunity cost for lands and facilities, 4% was added to the unit cost of ED and outpatient visits and 6% for an inpatient stay [53]. Unit costs for medical services were obtained for the financial year 2013–2014 to concord with the study timeframe.

Costs from the patient perspective included those paid by patients to receive health services such as the portion of drug costs not covered by the provincial drug plan and self-reported psychotherapy sessions. In previous studies, loss of productivity has been assessed by time lost to perform paid [55–57] and unpaid work [14, 58, 59]. In this study, indirect costs related to loss of productivity for unpaid time lost due to consultations and health care services used for all causes were also considered. The importance to account for unpaid work in economic evaluations has previously been highlighted in Canadian and other guidelines on economic evaluations [48, 60]. The loss of productivity was estimated at two hours for an outpatient visit, 18.4 h for an ED visit and eight hours for a day of hospitalization [14, 61]. The number of hours for an ED visit was determined by the mean wait time on a stretcher for all causes in Quebec in 2012–2013 [61]. Loss of productivity to receive health care services was calculated by multiplying the number of hours by the provincial minimum wage in 2013 [62]. Stephens and Joubert [63] estimated an hourly rate to account for loss of productivity for unpaid work. This hourly rate was similar to the minimum wage in Quebec in 1998 and therefore, we used the minimum wage in 2013 (hourly rate of 10.15$ CAD) [62] to estimate the loss of productivity for our sample of older adults who were mostly retired (96%) at the time of the study.

Total cost from the restricted societal perspective was obtained by adding the total costs obtained from the health system and patient perspectives [48]. Table 1 includes a detailed description of data sources and unit costs.

**Table 1:** Description and estimation of health care costs

Type of costs		Description and type of costs included	Data sources	Unit costs
**Health care system perspective**				
**Emergency department (ED)**		− Direct and indirect medical costs − General costs − Opportunity costs (4%) − Security and maintenance (9.7%) − Depreciation costs for buildings (10%)	RAMQ and MED-ECHO medical database Activity reports (AS-471) [51] Rosenheck et al. [53] (opportunity costs) Vasiliadis et al. [3] (security and maintenance) Government of Canada, [54] (depreciation costs)	$399 per visit
**Outpatient**	Mental health visits - local community service center			$80 per visit + $1417 per patient per year
	Mental health visit - outpatient clinic			$80 per visit + $792 per patient per year
	General medical visits in outpatient settings – not mental health related			$130 per visit
**Inpatient**	Mental health inpatient stay	− Direct and indirect medical costs − General costs − Opportunity costs (6%) − Security and maintenance (9.7%) − Depreciation costs for buildings (10%)		$485 per diem + $644 per hospitalization
	General inpatient stay			$593 per diem + $644 per hospitalization
	Day surgeries			$1900 per surgery
**Physician billing fees**		− Physician fees billed to the RAMQ	RAMQ medical database	
**Medications**		− Portion paid by the provincial public drug plan	RAMQ pharmaceutical database	
**Patient perspective**				
**Psychotherapy sessions in private practice**		− Costs paid by patients	ESA-Services questionnaire at baseline and follow-up	
**Medications**		− Portion paid by patients	RAMQ pharmaceutical database	
**Loss of productivity**	ED visit	− 18.4 h for an ED visit	Vasiliadis et al. [14] Stephens and Joubert [63] *Le Commissaire de la Santé et du Bien-Être* [61] Demers, 2018 [62] (Minimum wage: 10.15$ CAD)	$187 per visit
	Outpatient visit	− 2 h for an outpatient visit		$20 per visit
	Hospitalisation	− 8 h for a day of hospitalisation		$81 per day of hospitalisation

### Analyses

Among the analytic sample, 33 (9%) participants had miss ing data on the social and material deprivation indexes. Based on a procedure for categorical variables, these factors were imputed using the variables age and sex [64]. A total of 25 (7%) participants had missing data on psychological distress and anxiety symptom severity. These variables were imputed using the following procedure. Missing items for older adults who responded to at least 6 out of 10 questions of the K10, were replaced by the participant mean score on those items. For participants who answered at least 4 out of 7 questions related to the GAD-7, missing data were replaced by the participant mean score on those items. For participants who did not answer the minimum required items at baseline, the total score at follow-up was used if available.

Descriptive and bivariate analyses were conducted to describe the analytic sample and assess the association between receipt of minimally adequate treatment and study variables. Potential confounding factors that were significantly associated with the outcome variable, total societal costs, with a threshold of *p* < 0.10 were included in the multivariable models. These variables were also used to create a propensity score to estimate the probability of receiving minimally adequate treatment. This procedure is recommended in observational studies to minimize the effect of selection bias associated with receipt of treatment and potential confounding factors when the treatment received is not randomly assigned [65]. The inverse probability was used as a weight in unadjusted and adjusted cost analyses to control for treatment selection bias. Cost data are usually asymmetrically distributed as the majority of patients generate relatively low costs. The Kolmogorov-Smirnov and the Box tests were conducted to assess the normality and homoscedasticity of residuals, which showed that normality and homoscedasticity postulates were not respected. Generalized linear models with a gamma distribution and log link adjusting for potential confounders were carried out to assess the costs associated with receipt of minimally adequate treatment. Sensitivity analyses were conducted to account for the costs associated with health care use in the three years preceding the baseline interview. SPSS 27.0 and SAS 9.4 were used to conduct the bivariate and multivariable analyses and create the propensity score. Wald chi-square estimates are presented with their *p*-value.

## Results

The characteristics of the study sample are presented in Table 2. Overall, 39% of participants received minimally adequate treatment (i.e. pharmacologic and follow-up or psychological). Older adults with depression or an anxiety disorder receiving minimally adequate treatment reported a higher number of chronic physical diseases (5.19 vs 4.24, *p* = 0.001), higher psychological distress (24.46 vs 19.39, *p* < 0.001) and anxiety symptom severity (7.07 vs 5.25, *p* < 0.001) than those not receiving minimally adequate treatment. Participants that received minimally adequate treatment reported lower HRQOL (66.80 vs 72.20, *p* = 0.010) and satisfaction with life (18.51 vs 20.74, *p* < 0.001) at baseline than patients who did not receive adequate treatment.

**Table 2:** Characteristics of the overall analytic sample and according to the quality of care received

	Overall sample (***n*** = 346^a^)	Missing data	Receipt of minimally adequate mental health care		***p***-value
			Yes (***n*** = 134^a^)	No (***n*** = 212^a^)	
	n (%)				
*Individual factors*					
Type of CMD					
Depression alone	46 (12.9)	0 (0)	20 (14.4)	26 (11.9)	**< 0.001**
Anxiety disorder alone	274 (76.5)		92 (66.2)	182 (83.1)	
Both	38 (10.6)		27 (19.4)	11 (5.0)	
Sex					
Female	247 (69.0)	0 (0)	102 (73.4)	145 (66.2)	0.153
Male	111 (31.0)		37 (26.6)	74 (33.8)	
Education					
0–7 years	104 (29.2)	2 (0.6)	36 (25.9)	68 (31.3)	0.271
≥ 8 years	252 (70.8)		103 (74.1)	149 (68.7)	
Marital status					
Married/living with a partner	199 (55.6)	0 (0)	70 (50.4)	129 (58.9)	0.113
Not married/living with a partner	159 (44.4)		69 (49.6)	90 (41.1)	
Material deprivation index					
1 (lowest deprivation)	67 (19.1)	7 (2.0)	31 (22.5)	36 (16.9)	0.764
2	73 (20.8)		27 (19.6)	46 (21.6)	
3	67 (19.1)		26 (18.8)	41 (19.2)	
4	72 (20.5)		26 (18.8)	46 (21.6)	
5 (higher deprivation)	72 (20.5)		28 (20.3)	44 (20.7)	
Social deprivation index					
1 (lowest deprivation)	60 (17.1)	7 (2.0)	21 (15.2)	39 (18.3)	0.218
2	60 (17.1)		17 (12.3)	43 (20.2)	
3	58 (16.5)		27 (19.6)	31 (14.6)	
4	83 (23.7)		37 (26.8)	46 (21.6)	
5 (higher deprivation)	90 (25.6)		36 (26.1)	54 (25.3)	
	**Mean (SD)**	**n (%)**	** Mean (SD) **		***p*-value**
Age	73.52 (5.72)	0 (0)	73.43 (5.58)	73.58 (5.82)	0.811
Cognitive status (22–30)	28.10 (1.81)	0 (0)	28.10 (1.70)	28.10 (1.88)	0.999
Number of chronic diseases (0–17)	4.61 (2.55)	0 (0)	5.19 (2.88)	4.24 (2.25)	**0.001**
Psychological distress (10–50)	21.35 (7.46)	12 (3.4)	24.46 (7.89)	19.39 (6.47)	**< 0.001**
Severity of anxiety symptoms (0–21)	5.95 (3.77)	12 (3.4)	7.07 (3.98)	5.25 (3.45)	**< 0.001**
Social support (0–3)	2.76 (0.58)	0 (0)	2.75 (0.58)	2.77 (0.58)	0.708
Number of daily hassles (0–32)	10.55 (7.06)	0 (0)	11.28 (6.61)	10.09 (7.30)	0.120
Health-related quality of life (0–100)	70.10 (19.39)	0 (0)	66.80 (18.66)	72.20 (19.59)	**0.010**
Satisfaction with life (5–25)	19.88 (4.50)	2 (0.6)	18.51 (4.93)	20.74 (3.99)	**< 0.001**
Patients’ beliefs – mental health treatment (1–5)	3.90 (1.20)	20 (5.6)	3.28 (1.01)	3.28 (0.93)	0.969
Confidence in doctor to help with mental health issues (1–5)	3.28 (0.96)	17 (4.7)	3.82 (1.20)	3.95 (1.21)	0.327
Perceived adequacy of care for mental health problems (1–5)	3.92 (1.28)	45 (18.2)	3.89 (1.26)	3.95 (1.30)	0.689
Number of days alive in the three-year period	1073.20 (98.51)	0 (0)	1080.67 (81.21)	1068.47 (107.98)	0.225
*Health system factor*	**n (%)**				***p*-value**
Type of primary care practices					
Small clinics (< 3 GP)	140 (40)	10 (2.8)	52 (38.8)	88 (41.1)	0.668
Large clinics (≥ 3 GP)	208 (60)		82 (61.2)	126 (58.9)	

*CMD* Common mental disorders, *GP* general practitioners, *SD* Standard deviation

Significant results are in bold (*p* < 0.05)

^a^Sample sizes vary according to study variables. The lowest sample sizes are presented

The association between study variables and unweighted total societal costs are presented in Table 3. With a threshold of *p* < 0.10, higher total societal costs were associated with lower level of education (B: 0.204, SE: 0.112, *p* = 0.067), higher levels of material and social deprivation, older age (B: 0.020, SE: 0.009, *p* = 0.033), lower cognitive functioning (B: -0.058, SE: 0.028, *p* = 0.039), a higher number of chronic diseases (B: 0.098, SE: 0.020, *p* < 0.001), higher level of psychological distress (B: 0.014, SE: 0.007, *p* = 0.048), lower health-related quality of life (B: -0.013, SE: 0.003, *p* < 0.001) and a lower number of days contributing to the study period (B: -0.001, SE: 0.001, *p* = 0.006). A total of 338 participants had a complete dataset for these study variables and therefore, were included in the multivariable models.

**Table 3:** Bivariate associations between study variables and three-year societal costs

	Beta (SE)	Wald χ2	***p***-value
*Individual factors*			
Minimally adequate treatment			
Yes	0.365 (0.102)	12.748	**< 0.001**
No			
Sex			
Female	−0.126 (0.109)	1.330	0.249
Male			
Education			
0–7 years	0.204 (0.112)	3.345	0.067
≥ 8 years			
Marital status			
Married/living with a partner	−0.029 (0.102)	0.080	0.777
Not married/living with a partner			
Material deprivation index			
1 (lowest deprivation)		REF	
2	0.299 (0.162)	3.386	0.066
3	0.104 (0.166)	0.393	0.531
4	0.370 (0.163)	5.152	**0.023**
5 (higher deprivation)	0.252 (0.163)	2.395	0.122
Social deprivation index			
1 (lowest deprivation)		REF	
2	−0.177 (0.174)	1.032	0.310
3	0.059 (0.176)	0.113	0.737
4	0.329 (0.162)	4.134	**0.042**
5 (higher deprivation)	0.033 (0.159)	0.043	0.836
Age	0.020 (0.009)	4.559	**0.033**
Cognitive status (22–30)	−0.058 (0.028)	4.274	**0.039**
Number of chronic diseases (0–17)	0.098 (0.020)	24.632	**< 0.001**
Psychological distress (10–50)	0.014 (0.007)	3.910	**0.048**
Severity of anxiety symptoms (0–21)	0.008 (0.013)	0.369	0.544
Social support (0–3)	−0.089 (0.084)	1.133	0.287
Number of daily hassles (0–32)	−0.001 (0.008)	0.024	0.877
Health-related quality of life (0–100)	−0.013 (0.003)	25.300	**< 0.001**
Satisfaction with life (5–25)	−0.017 (0.011)	2.482	0.115
Patients’ beliefs – mental health treatment (1–5)	−0.063 (0.054)	1.344	0.246
Confidence in doctor to help with mental health issue (1–5)	0.036 (0.042)	0.751	0.386
Perceived adequacy of care for mental health problems (1–5)	−0.018 (0.043)	0.174	0.677
Number of days alive during the three-year period	−0.001 (0.001)	7.568	**0.006**
*Health system factor*			
Type of primary care practices			
Small clinics (< 3 GP)	0.019 (0.105)	0.034	0.853
Large clinics (≥ 3 GP)	REF		

Results obtained with generalized lineal models using gamma distribution and log link

*GP *general practitioners,* SE* standard error

Significant results are in bold (*p* < 0.05)

Weighted unadjusted and adjusted cost estimates associated with receipt of minimally adequate treatment for depression and anxiety disorders are presented in Table 4. After adjusting for socio-demographic and clinical factors, receipt of minimally adequate treatment was associated with higher total health system costs (Δ$5752, *p* < 0.001) and expenses from the patient perspective (Δ$536, *p* = 0.001) and the societal perspective (Δ$6266, *p* < 0.001) compared to older adults not receiving minimally adequate treatment. From the health system perspective, receipt of minimally adequate treatment was associated with higher costs related to ED visits (Δ$457, *p *= 0.001), psychiatric inpatient stays (Δ$856, *p* < 0.001), outpatient mental health visits (local community service center: Δ$1, *p* < 0.001 and outpatient clinic: Δ$10, *p *< 0.001), outpatient visits for other diagnoses (Δ$568, *p* < 0.001) and overall, total inpatient (Δ$2564, *p* = 0.025) and outpatient costs (Δ$620, *p* < 0.001). Older adults receiving minimally adequate treatment also incurred higher medication costs paid by the provincial drug plan (Δ$1243, *p* = 0.002) and physician fees paid out (Δ$1224, *p* < 0.001). From the patient perspective, receipt of minimally adequate treatment was associated with higher costs due to loss of productivity related to ED (Δ$213, *p* < 0.001) and outpatient visits (Δ$89, *p* < 0.001). Not receiving minimally adequate treatment was associated with higher costs for self-reported psychotherapy sessions (Δ$1, *p* = 0.049) as compared to receiving minimally adequate treatment.

**Table 4:** Three-year total weighted costs ($) associated with adequacy of mental health care in older adults with an anxiety disorder or depression

	Mean cost unadjusted ***n*** = 338		Wald χ2	***p***-value	Mean cost adjusted *n* = 338^a^		Wald χ2	***p***-value
	Receipt of minimally adequate care				Receipt of minimally adequate care			
	Yes	No			Yes	No		
***Health system perspective***								
Emergency department (ED) visits	1011	610	9.655	**0.002**	985	528	10.859	**0.001**
Inpatient								
Hospitalization stay – Mental health	2412	1327	6.185	**0.013**	969	113	36.456	**< 0.001**
Hospitalization stay – Other diagnoses	3515	3041	0.434	0.510	3712	2330	2.795	0.095
Day surgeries	1208	674	8.060	**0.005**	886	553	3.575	0.059
** Total inpatient**	7135	5042	3.702	0.054	6643	4079	5.032	**0.025**
Outpatient								
Outpatient visits – Mental health, local community service center	29	1	1341.819	**< 0.001**	2	1	50.665	**< 0.001**
Outpatient visits – Mental health, outpatient clinic	24	7	86.320	**< 0.001**	12	2	104.651	**< 0.001**
Outpatient visits – Other diagnoses	1810	1177	20.585	**< 0.001**	1749	1181	14.383	**< 0.001**
** Total outpatient**	1863	1185	25.255	**< 0.001**	1810	1190	18.172	**< 0.001**
Medications – paid by the public plan	8038	4869	45.169	**< 0.001**	6016	4773	9.500	**0.002**
Physician fees	5187	3803	23.327	**< 0.001**	4987	3763	17.777	**< 0.001**
** Total cost**	23,234	15,509	32.368	**< 0.001**	20,700	14,948	20.400	**< 0.001**
***Patient perspective***								
Psychotherapies	3	4	3.797	0.051	1	2	3.859	**0.049**
Lost in productivity								
ED visits	474	286	10.962	**< 0.001**	461	248	12.420	**< 0.001**
Inpatient stay	790	570	2.895	0.089	696	428	3.624	0.057
Outpatient visits	283	184	24.539	**< 0.001**	274	185	17.315	**< 0.001**
Medications – paid by the patient	1733	1599	2.736	0.098	1684	1612	0.711	0.399
** Total cost**	3283	2643	16.931	**< 0.001**	3166	2630	11.869	**0.001**
***Societal perspective***								
** Total cost**	26,517	18,152	32.572	**< 0.001**	23,888	17,622	20.666	**< 0.001**
**Sensitivity analysis**^b^								
***Societal perspective***								
** Total cost**	26,517	18,152	32.572	**< 0.001**	19,657	17,031	5.662	**0.017**

Significant results are in bold (*p* < 0.05)

^a^Adjusted for: age, education, social and material deprivation indexes, cognitive functioning, number of chronic diseases, psychological distress, health-related quality of life and the number of days alive during the study period

^b^Further adjusted for societal costs incurred during the three years preceding the baseline interview

Sensitivity analyses showed that receipt of minimally adequate treatment for CMD, as opposed to not, remained associated with higher three-year health care costs from the restricted societal perspective (Δ 2626$, *p* = 0.017) after further adjusting for societal health care costs incurred in the three years preceding the interview.

## Discussion

The current study aimed to report on the health system, patient and societal costs associated with receipt of minimally adequate treatment in primary care older adults with depression and anxiety disorders. This study adds to the literature by presenting results based on health survey and administrative data minimizing the potential for information bias and allowing the control of important socio-demographic and clinical factors.

Individuals receiving minimally adequate treatment had a higher number of chronic diseases and increased severity of depression and anxiety symptoms. These results concord with previous research in adults [28, 66, 67]. These individuals also reported lower HRQOL and satisfaction with life at baseline. Previous studies in clinical settings showed that adults receiving guideline-concordant care for depression and panic disorder had lower HRQOL [68, 69].

The current findings showed that receipt of minimally adequate treatment was associated with higher unadjusted and adjusted three-year costs from the restricted societal perspective averaging $8365 and $6266, or $2788 and $2089 per year, respectively.

When breaking down the costs from the health system, patient and societal perspectives, receipt of minimally adequate treatment was associated with higher adjusted costs averaging $5752, $536, $6266, respectively. Reports on the topic in the literature have not been consistent [10–13, 70]. In a Dutch primary care study of adult patients, Prins et al. [12] reported that receiving guideline-concordant treatment for depression and anxiety was not significantly associated with total annual direct costs, which included costs for primary and secondary care, medication and supportive care. A Canadian study based on administrative data showed that adults with depression receiving the lowest and highest levels of guideline-concordant care incurred the highest costs [10]. Care received was categorized from 0 (did not receive an antidepressant) to 5 (adequate antidepressant type, adequate dose and duration) [10]. This last Canadian study included adults who received a clinical diagnosis of depression and were covered under Quebec’s provincial drug plan. It was previously suggested that mental disorders are under-reported in administrative da tabases in Canada [71], which could suggest that the sample of patients with diagnosed depression may have more severe symptoms. Compared to the latter, the current study was conducted in older adults with multiple physical chronic diseases suggesting the presence of comorbidities and more complex clinical profiles overall. 

After adjusting for potential confounders, differences in health system costs observed were driven by inpatient stays for a psychiatric reason, ED and outpatient visits, medication costs and physician fees paid out. The three-year adjusted costs incurred for inpatient stays due to mental disorders were nearly 8 times higher in patients receiving minimally adequate treatment. In Robinson et al.’s [11] retrospective study reporting on unadjusted 6-month inpatient costs for depression, there was no association with adherence to guidelines in adults with depression. Another study on data from private insurers reported that, among patients with a history of cardiovascular disease, outpatient visits were higher in adults receiving inadequate antidepressant treatment for depression reaching $765 US [13]. The differing results may in part be due to the fact that these latter studies included health insurance claims for adults with depression covered by a private insurer in the United States, which may also differ from the present cohort on socio-economic and health status [11, 13].

The current results also showed that receiving minimally adequate treatment was significantly associated with higher costs related to ED visits over the three-year period. This showed that despite receiving adequate outpatient follow-up care, older adults were still more likely to consult ED services. In fact, a large population-bas ed study similarly showed a higher number of ED visits in individuals with mental disorders also consulting on an outpatient basis, and in those receiving adequate follow-up care [72]. Longitudinal studies should focus on better describing the health service trajectories (e.g. ED visits and inpatient stay in relation to the timing of outpatient visits) of older adults with common mental disorders and the demographic and clinical profiles of individuals incurring higher costs for ED visits and hospitalizations despite receiving adequate outpatient follow-up care. Future studies also need to look more closely at continuity of care when assessing criteria in defining adequate treatment.

The results also suggest that participants receiving minimally adequate treatment for CMD have more severe symptoms and therefore, might have better access and follow-up care resulting in higher health care costs. Additional analyses showed that participants who received minimally adequate treatment had increased ED visits in the first two years, but not the third year, and hospitalizations in the first year but not in the second and third year, suggesting that health care use is mainly associated with the episode of a CMD lasting on average 23 months [18]. Further, sensitivity analyses were conducted to account for past health care use. The results similarly showed that after further adjusting for past health care costs, receipt of minimally adequate treatment remained significantly associated with higher societal costs.

The costs due to loss of productivity related to ED and outpatient visits and total costs from the patient perspective were higher in older adults receiving minimally adequate treatment. Only one study has looked at the indirect costs associated with guideline-concordant care for depression and anxiety disorders in adults aged 18 to 65 years old [12]. This study did not report any significant differences in terms of costs related to loss of productivity for paid work and total indirect costs between participants who received and did not receive guideline-concordant treatment [12]. In that study, loss of productivity and efficiency for paid work was considered [12], while in the current study, loss of productivity for unpaid work related to time lost in general while receiving medical care in ambulatory and inpatient settings was considered. These findings suggest the importance in differentiating costs related to loss of productivity due to employed work (absenteeism, presenteeism) and non-paid work. Further, few were the participants that reported receiving psychotherapy resulting in low incurred costs for these services. In Quebec, psychotherapy sessions provided by mental health professionals are not covered universally under the provincial public health insurance plan limiting access to these services for individuals without private insurance that are faced with long wait times in the public sector [73–75]. In Quebec, 90% of older adults aged 65 years and over are covered under the provincial public drug plan [76] suggesting that they do not have a private health insurance. This finding may suggest that older adults in the current study had limited access to psychotherapy, which may have influenced the proportion of those receiving minimally adequate treatment for depression and anxiety disorders.

The results of the current study were not always consistent with previous findings. Differences may be in part attributed to study populations, definition and categorization of adequacy of care and the different costs considered. Most studies included patients with depression [10, 11, 70], while only one included a sample where 43% had an anxiety disorder only and 36% had both depression and an anxiety disorder [12]. In the current study, the sample included 76% of individuals with an anxiety disorder alone and 11% had both depression and anxiety. The different clinical and psychiatric symptom profiles may in part explain the associations observed between receipt of minimally adequate treatment and societal costs. Further, none of the previous studies were conducted in older adults and included healthier populations with respect to physical comorbidities. The definition of minimally adequate treatment also varied between studies. Only Prins et al. [12] considered psychological support in the definition of guideline-concordant care while others only included quality indicators for antidepressant treatment [10, 11, 13]. Further, Knapp and Wong [77] suggest that the economic burden associated with mental disorders given their chronicity should be observed over longer periods. The 3-year observation period in assessing the long-term costs associated with receipt of minimally adequate treatment in the current study was long enough to be able to capture the episodic nature of depression and anxiety disorders where an episode lasts on average 23 months in older adults [18]. Most studies however had evaluated costs on a short-term period ranging between 6 and 12 months [10–12].

The current study suggests that older adults who received minimally adequate treatment, as opposed to those who did not, incurred higher costs from the restricted societal perspective. Studies reporting on the association between receipt of adequate treatment and short- and long-term outcomes are inconsistent [9, 78, 79]. Primary care patients who received adequate care for depression had an improvement in their symptoms after a year [79] and reported better satisfaction with life after three years [9]. More research is needed in epidemiologic settings to better understand the effects of receiving adequate treatment for CMD and this by studying societal costs with respect to outcomes such as quality of life gained due to treatment. This will contribute in better informing policy makers on the allocation of resources.

Potential savings to society by treating CMD have not been considered in studies. In fact, longer studies could inform on future saving to society associated with receiving minimally adequate treatment. For example, reduced health system and indirect costs associated with increased quality of life and functioning may be important to consider given that a previous Canadian study reported that loss in quality of life was responsible for 55% of the societal burden associated with mental disorders [80]. Receiving minimally adequate treatment for CMD in older adults was also associated with reduced all-cause mortality [81]. Therefore, indirect costs associated with avoided premature death (e.g. suicide, reduced life expectancy due to mental illness) [82–86] may also be important to consider in future studies in older adult populations.

The current findings should be interpreted with the following considerations. First, the analytic sample included older adults recruited in different type of primary care clinics that included private offices with three and more physicians, less than three physicians, general medicine groups and community local health and social service centers. There was no difference between type of primary care setting recruited in and receipt of minimally adequate treatment, therefore limiting the potential selection bias. Second, older adults covered under the provincial public drug plan were included in the analytic sample. Additional analyses showed that participants with a common mental disorder who were and were not covered under the provincial drug plan had similar characteristics according to age, sex, education, area level deprivation indexes, cognitive functioning, number of chronic physical disorders and severity of depression and anxiety symptoms. However, older adults not covered by the public drug plan were more likely to be married or live with a partner. Third, 10% of the sample died in the three-year follow-up period. To limit the potential selection bias, we included these participants in the analytic sample, and the number of days the participants contributed to the study period was used to estimate the propensity score and adjust multivariable analyses. Further analyses did not show a difference in deaths in the adequate and non-adequate group, limiting the possible selection bias. Fourth, the RAMQ administrative databases are for billing purposes and errors in billing and diagnostic codes could have influenced the classification of individuals. Furthermore, physicians are not required to enter diagnoses to be paid, which could have led to the underestimation of costs related to mental health reasons. Fifth, considering that we did not have access to medical files and physician notes on medical history and treatment preference, we included all medications recommended to treat depression and anxiety disorders. Although, Canadian clinical guidelines account for severity of illness, given the data available only minimal indicators of adequate treatment were used to assess receipt of minimally adequate treatment. To overcome this potential bias, severity of psychological distress and anxiety symptoms were considered in the calculation of the propensity score and were used to adjust the analyses. In the current study, the use of anxiolytics was included in the definition of adequate pharmacological treatment for anxiety disorders. According to the Beers criteria, benzodiazepines are only recommended for specific disorders (e.g. severe generalized anxiety disorder) in older adults [87]. Additional analyses showed that when excluding the delivery of a benzodiazepine from the definition of receipt of adequate treatment, the results did not change. One also has to consider that pharmacotherapy retained from the RAMQ pharmaceutical database includes information on medications delivered to patients and not to physician prescribing or to patient adherence. Sixth, psychotherapy sessions were only measured in the six months before the interview, which could have underestimated the patient costs related to these services. Considering that costs related to these services were similar between participants who received and did not receive adequate treatment, the potential information bias is more likely non-differential. Further, in this study sample, 22 participants were not covered by the public drug plan for the entire three-year period. These participants were probably covered by a private insurance and insurance costs were not included in this study. Additional analyses revealed that the proportion of participants who were not covered under the public drug plan were just as likely to receive minimally adequate treatment, thus limiting the potential information bias resulting in conservative estimates. Further, wait time to access psychotherapy with mental health professionals in the public sector was not included in this study. Seventh, given that the definition of minimally adequate treatment included the prescription of drugs, follow-up medical visits, and the presence of psychotherapy sessions, older adults who received minimally adequate treatment were more likely to incur higher costs in the first year after the baseline interview. Additional analyses showed that receiving minimally adequate treatment was associated with greater ED visits for the first two years, hospitalizations for the first year and outpatient visits for the three years following the interview suggesting the presence of a differential information bias. Eight, although propensity score analysis was used to control for the potential selection bias and confounding by indication associated with receipt of minimally adequate treatment, in the absence of a controlled trial, the associations observed in the current study can’t be interpreted as reflecting causality. Finally, the analytic sample came from a convenience sample of French-speaking older adults aged 65 years and over consulting in primary care in one administrative region in Quebec, Canada. The results of this study may not be generalizable to older adults living in different health system settings.

Finally, the present study contributed to the literature by assessing the three-year societal costs associated with receiving minimally adequate pharmacological and psychological treatment for depression and anxiety disorders in an older population. The findings showed that receipt of minimally adequate treatment was associated with higher three-year health care costs from the societal perspective compared to older adults not receiving adequate treatment. Receipt of minimally adequate treatment was associated with increased ED visits in the first two years, but not the third year, and hospitalizations in the first year but not in the second and third year. Future research should focus on better documenting during longer follow-up, beyond the average duration of episodes of depression and anxiety, health service utilization and related costs to better capture and inform on the return when investing in the quality of mental health services and treatment of common mental disorders in older adults.

## Data Availability

The authors are not legally authorised to share or publicly publish linked survey and health administrative data due to privacy or ethical restrictions related to the use of administrative provincial health data. Participants were not requested to give informed consent for data sharing. The province of Québec’s ‘Commission d’Accès à l’Information’ gave approval to merge these datasets. Questions should be addressed to the research ethics committee of the CIUSSS de l’Estrie-CHUS.

## Abbreviations

CMD: Common mental disorders
ED: Emergency department
ICD: International classification of diseases
GAD-7: 7-item Generalized Anxiety Disorder
GP: General practitioner
HRQOL: Health-related quality of life
K10: 10-item Kessler Psychological Distress Scale
MED-ECHO: Maintenance et exploitation des données pour l’étude de la clientèle hospitalière
MMSE: Mini-Mental State Examination
RAMQ: Régie de l’Assurance Maladie du Québec
SD: Standard deviation
SE: Standard error
YLD: Years lived with disability
